# The Influence of Mixed Activators on Ethylene Polymerization and Ethylene/1-Hexene Copolymerization with Silica-Supported Ziegler-Natta Catalyst

**DOI:** 10.3390/molecules15129323

**Published:** 2010-12-16

**Authors:** Nichapat Senso, Supaporn Khaubunsongserm, Bunjerd Jongsomjit, Piyasan Praserthdam

**Affiliations:** 1 Center of Excellence on Catalysis and Catalytic Reaction Engineering, Department of Chemical Engineering, Faculty of Engineering, Chulalongkorn University, Bangkok 10330, Thailand; 2 PTT Research and Technology Institute, PTT Public Company Limited, Wangnoi, Ayuthaya 13170, Thailand

**Keywords:** Ziegler-Natta catalyst, silica support, polyethylene, mixed activator, ethylene polymerization

## Abstract

This article reveals the effects of mixed activators on ethylene polymerization and ethylene/1-hexene copolymerization over MgCl_2_/SiO_2_-supported Ziegler-Natta (ZN) catalysts. First, the conventional ZN catalyst was prepared with SiO_2_ addition. Then, the catalyst was tested for ethylene polymerization and ethylene/1-hexene (E/H) co-polymerization using different activators. Triethylaluminum (TEA), tri-*n*-hexyl aluminum (TnHA) and diethyl aluminum chloride (DEAC), TEA+DEAC, TEA+TnHA, TnHA+ DEAC, TEA+DEAC+TnHA mixtures, were used as activators in this study. It was found that in the case of ethylene polymerization with a sole activator, TnHA exhibited the highest activity among other activators due to increased size of the alkyl group. Further investigation was focused on the use of mixed activators. The activity can be enhanced by a factor of three when the mixed activators were employed and the activity of ethylene polymerization apparently increased in the order of TEA+ DEAC+TnHA > TEA+DEAC > TEA+TnHA. Both the copolymerization activity and crystallinity of the synthesized copolymers were strongly changed when the activators were changed from TEA to TEA+DEAC+TnHA mixtures or pure TnHA and pure DEAC. As for ethylene/1-hexene copolymerization the activity apparently increased in the order of TEA+DEAC+TnHA > TEA+TnHA > TEA+DEAC > TnHA+DEAC > TEA > TnHA > DEAC. Considering the properties of the copolymer obtained with the mixed TEA+DEAC+TnHA, its crystallinity decreased due to the presence of TnHA in the mixed activator. The activators thus exerted a strong influence on copolymer structure. An increased molecular weight distribution (MWD) was observed, without significant change in polymer morphology.

## 1. Introduction

Progress in catalyst technology has lead to the synthesis of a rich set of new polymers with different structures and performances to meet the progressive demands of modern industry and life [1,2,3,4,5,6,7,8,9,10,11,12]. Recently, branched polyethylenes such as linear low-density polyethylene (LLDPE) have grown in importance in industry because of the specific properties that can be obtained by varying comonomer content and polymerization conditions. The recent development of homogeneous single-site catalyst makes it possible to synthesize the copolymers with completely different structures and performances from traditional polyethylenes [13]. The ethylene/α-olefin copolymers obtained by metallocene catalysts show homogeneous comonomer distribution and narrow molecular weight distributions in comparison with those obtained with traditional Ziegler–Natta (ZN) catalysts [14]. The correlation between the structures of the ethylene/α-olefin copolymers obtained with ZN catalysts and their properties has been extensively studied [15,16]. However, easy methods to control polymer properties and catalytic activity in polymerization system are still of concern to the industry. 

One of the most important factors in ethylene and ethylene/α-olefin polymerization is the choice of alkyl aluminum used to control the activity and polymer characteristics. The alkyl aluminums are often added to the reactor during slurry polymerization with ZN catalyst and conventional supported metallocene/MAO catalyst to scavenge impurities. In polymerization systems, alkyl aluminums also act as activators responsible for the generation of active sites. There are many reports on the use of alkyl aluminum as activators in the polymerization of ethylene and ethylene/α-olefins using ZN catalysts [17,18,19,20,21,22,23]. Trialkyl aluminum compounds are usually preferred over the halogen-containing analogues because higher polymerization rates can be obtained with the former. Alkyl aluminums such as trimethyl aluminum (TMA), triethyl aluminum (TEA), tri-*n*-hexyl aluminum (TnHA) and triisobutyl aluminum (TiBA), as well as diethyl aluminum chloride (DEAC) have been used in olefin polymerizations [24,25,26,27,28,29,30,31]. In general, it has been found that an increase in size of the alkyl groups (C_n_H_2n+1_) up to approximately n = 11 has enhanced catalytic activity. The study by Wanke *et al*. [18] revealed that increasing the size of alkyl group with n < 11 produces an increase in the activity and when a very large alkyl groups (n = 18) was used, the catalytic activity was very low upon when using the ZN catalyst for ethylene polymerization. An exception to these general trends was observed by Nooijen [17]. His results showed a strong effect of activators (TEA, TiBA, TnOA and IPRA) diffusion on the rate of activation of MgCl_2_-supported Ziegler-Natta catalyst in slurry polymerization. At a constant ratio of activator to catalyst, the maximum activity depends on the diffusion of the activator. 

The properties of polymer, such as the morphology of polymer product particles [22], were also controlled by type of activator, the product molar mass distribution, the average catalyst activity, and the shape of the activity-time profiles [22,23]. Increasing the size of the ligands attached to aluminium atoms increased the average molar masses and resulted in narrower molar weight distributions of polyethylene [18]. Terano *et al.* [23] reported that the molecular weight distribution (MWD) of the polyethylene obtained from the functionalized SiO_2_-supported catalyst changed markedly from broad and multimodal to narrow and unimodal depending on the type of activator used. In the case of TEA, a broad trimodal MWD was observed, while for DEAC, the MWD of polyethylene was very narrow and unimodal [23]. In the case of poly[propylene-co-(7-methyl-1,6-otadiene)], the different activators produced polypropylene with a wide range of MWD [32]. 

Not only single activators are used in α-olefin polymerization, but also mixtures of alkyl aluminums are interesting subjects for improvement of the catalytic activity and polymer properties. Fan *et al.* [20] synthesized PE-PP copolymers using TEA, TIBA or TEA+TIBA mixtures as activators with MgCl_2_/SiO_2_/TiCl_4_/diester-ZN catalyst in a slurry polymerization process. Their results showed that the behaviors of the TEA/TIBA mixture in catalytic systems were not a simple superposition of those activated by the TEA or TIBA alone. When a 50:50 TEA+TIBA mixture was used, the copolymerization activity became the highest, and the yields of both systems were highly random copolymers. In their articles, rapid exchange between the alkyl groups in mixtures of TEA with the *iso*-butyl group in TIBA may be an important reason for the increase in catalytic activity and yields of both the random copolymer and the segmented copolymer parts, which were close to the highest level in PE-PP copolymer. 

In spite of these interesting results, the effect of mixed alkyl aluminum on catalytic activity of ethylene polymerization and ethylene/1-hexene copolymerization, and polymer properties has received little attention, even though it could be of crucial importance to successfully design and operate industrial polymerization processes. In the current study, the effect of various activator mixtures on activity, product morphology and molecular weight distribution of polyethylene and ethylene/1-hexene copolymere synthesized by the MgCl_2_/SiO_2_/TiCl_4_/THF-ZN catalyst was investigated. The obtained polymers were characterized by means of X-ray diffraction (XRD), gel permeation chromatography (GPC), differential scanning calorimetry (DSC), and nuclear magnetic resonance (^13^C-NMR) techniques.

## 2. Results and Discussion

### 2.1. Catalyst characterization

**Figure 1:**
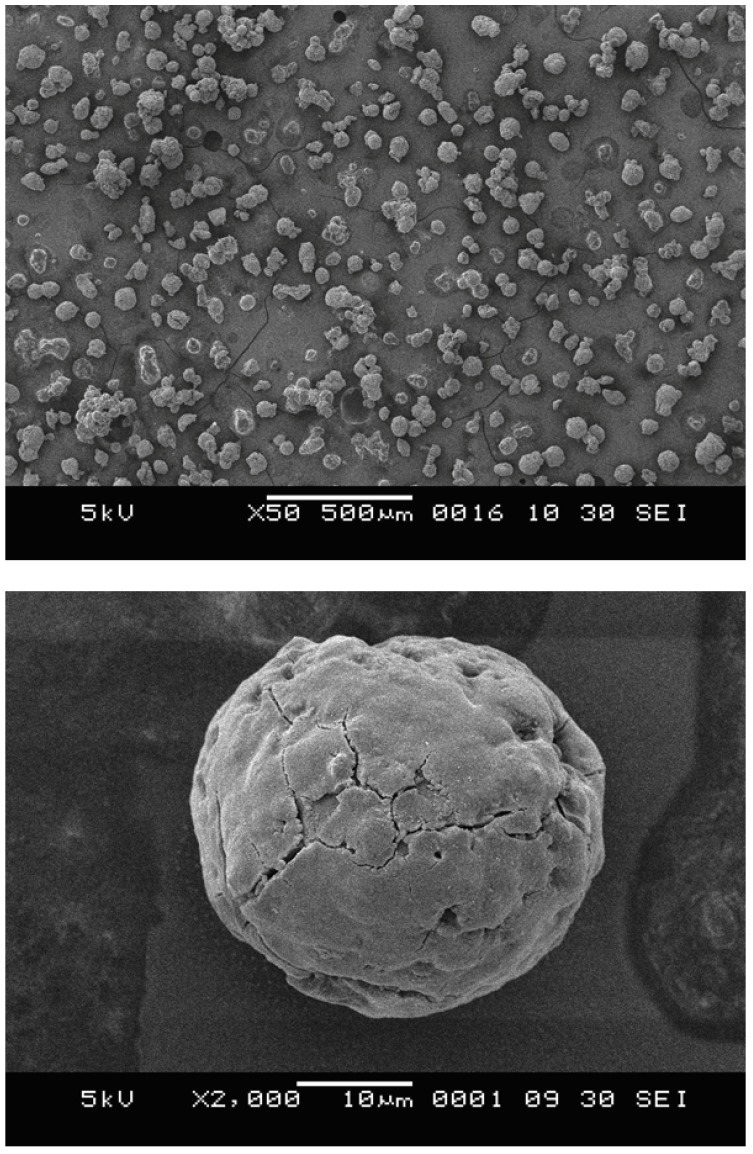
SEM micrographs of MgCl_2_/SiO_2_/TiCl_4_/THF-ZN catalyst.

In general, the MgCl_2_/SiO_2_/TiCl_4_/THF-ZN catalyst has been developed for an excellent morphology control of polymer particles under the fluidized bed reactor conditions [33,34]. Kim *et al.* [34,35] reported that catalyst characteristics such as the ratio of SiO_2_/MgCl_2_ had an influence on the shape and size of the MgCl_2_/SiO_2_/TiCl_4_/THF-ZN catalyst used for ethylene polymerization and ethylene/1-butene copolymerization. In this study, the MgCl_2_/SiO_2_/TiCl_4_/THF-ZN catalyst was prepared as described in the experimental part using a SiO_2_/MgCl_2_ molar ratio of 1:1. Based on this preparation, the presence of Ti content in catalyst is 2.33 wt% (ICP). The shape and size of the catalyst were observed by SEM as shown in Figure 1. The prepared catalyst exhibits a spherical shape and unimodal size distribution. Thus, the aggregated and melted form of catalyst particles seen in Figure 1 (top) are only present in a small amounts. These fractions possibly resulted from contact of catalyst particles with moisture and oxygen, when analyzed by SEM. The average diameter of obtained catalyst is approximately 30, µm as seen in Figure 1 (bottom). 

### 2.2. Ethylene homo-polymerization

**Figure 2:**
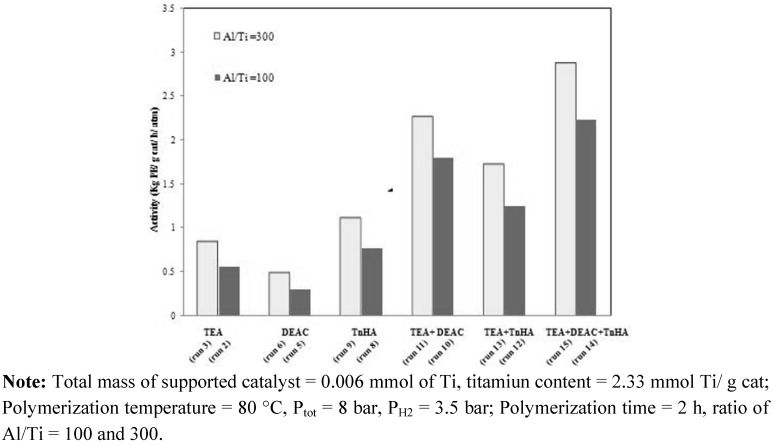
Effect of various activators on ethylene polymerization activity using MgCl_2_/SiO_2_/TiCl_4_/THF-ZN catalyst.

For commercial production of polyethylene, the MgCl_2_/SiO_2_-supported ZN catalyst exhibits high catalytic activity when TEA is used as activator. This activator provides the highest activity among other activators, including diethyl aluminum chloride (DEAC) and tri-*n*-hexyl aluminum (TnHA) [24]. The MgCl_2_/SiO_2_/TiCl_4_/THF-ZN catalyst shows high catalytic activity for ethylene polymerization. In this work, the effects of different types of alkyl aluminums including TEA, DEAC, TnHA and mixed alkyl aluminums on activity were investigated. The Al/Ti molar ratio was fixed at 100 and 300. The polymerization activities with various activators are shown in Figure 2 and Table 1. 

**Table 1:** Description of ethylene polymerization with various activators.

Run number		Activator^a^		Al/Ti	Activity^b^ (kg PE/ g cat/ h/ atm)
	TEA (mol%)	DEAC (mol%)	TnHA (mol%)		
1	100	-	-	100	0.53
2	100	-	-	100	0.55
3	100	-	-	300	0.83
4	-	100	-	100	0.32
5	-	100	-	100	0.30
6	-	100	-	300	0.48
7	-	-	100	100	0.78
8	-	-	100	100	0.76
9	-	-	100	300	1.10
10	50	50	-	100	1.79
11	50	50	-	300	2.25
12	50	-	50	100	1.25
13	50	-	50	300	1.71
14	33	33	33	100	2.23
15	33	33	33	300	2.86

^a^ Concentrations of TEA, DEAC and TnHA are 0.300 mmol/mL; ^b^ Ti concentration is 0.006 mmol/mL.

Considering the single activators, the catalytic activity was the highest when TnHA was employed, whereas DEAC exhibited the lowest activity. A similar trend was observed with regards to change in Al/Ti ratio. These results were also consistent with those reported by Lynch *et al.* [18] and Hammawa *et al.* [22]. For trialkyl aluminum with n < 11, the activity increases with the size of alkyl group [22]. It was observed that DEAC was a less effective activator than TnHA, as reported by Haward *et al.* [24]. They explained that there was an optimal ligand size for producing maximum catalyst activity. Based on this work, by mixing activators having different sizes of alkyl groups, the catalytic activity of each system was in the order of; TEA+DEAC+TnHA > TEA+DEAC > TEA+TnHA, as listed in Table 1. In addition, the mixed DEAC+TnHA (50:50) was also tested for ethylene polymerization (data not shown), but it gave low activity than seen for DEAC or TnHA alone. Hence, the catalytic activity can be enhanced by a factor of three when the suitable mixed activators are employed. 

**Scheme 1:**
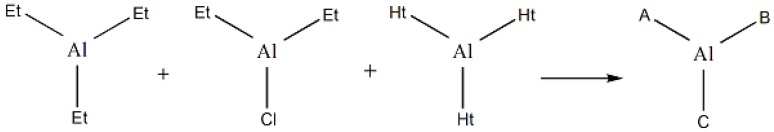
The possible formation of new alkyl groups after mixing (A, B and C refer to ethyl, *n*-hexyl and Cl).

These results can be described by: (i) each type of alkyl aluminum has different reducing ability towards the catalyst, and hence produces different types of active sites [22], (ii) the optimal ligand size for producing maximum catalyst activity can be obtained by mixing the various types of activator [24], and (iii) it is related to the real mechanistic roles of alkyl aluminum activator in the formation of the active site in heterogeneous Ziegler-Natta catalysis, using either a monometallic or bimetallic active site model. Besides, changes in forms of mixed alkyl aluminums during polymerization were also a possible reason. To determine the rapid exchange of alkyl groups in the mixed alkyl aluminums, ^1^H-NMR measurements were performed by Hatada *et al.* [36]. They found that the ^1^H-NMR spectrum of the mixture of TEA and DEAC at room temperature displayed a rapid intermolecular exchange of ethyl groups. However, a new ^1^H-NMR signal occurred upon the measurement at low temperature. In Scheme 1, we propose that the formation of new alkyl groups after mixing various alkyl groups may occur through various possible mechanisms. It should be noted that besides the formation of Al-ABC (as shown), other forms of mixed activator such as Al-ACC, Al-BBC, Al-BCC, and so on can occur. 

**Scheme 2:**
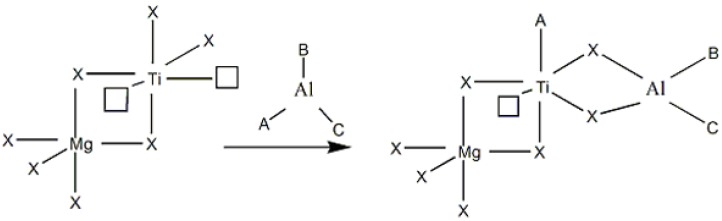
Suggested mechanism of active site formation activated by new alkyl aluminum type (X: -Cl; A, B or C: ethyl, n-hexyl or Cl; □: coordination vacancy).

The new alkyl groups may be suitable for adding more steric hindrance to the surface of catalyst enhancing the performance of ethylene to occupy the active species, as illustrated in Scheme 2. Moreover, the formation of catalyst might have a reducing ability to produce active sites for ethylene polymerization.

### 2.3. Ethylene/1-hexene copolymerization

**Figure 3:**
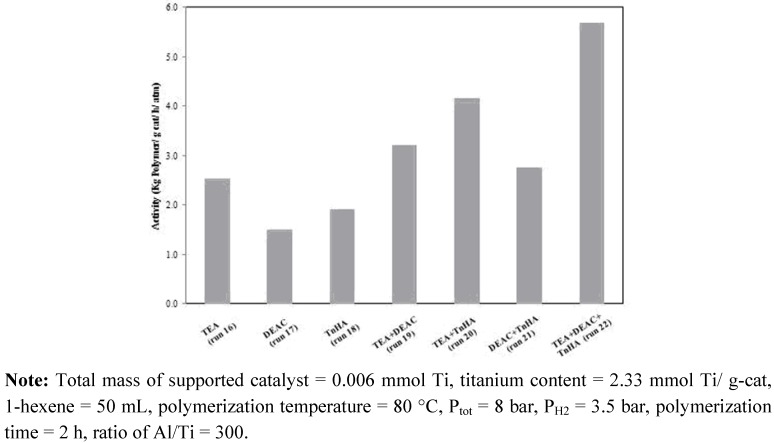
Effect of mixed activators on catalytic activity of MgCl_2_/SiO_2_/TiCl_4_/THF-ZN catalyst for ethylene/1-hexene copolymerization.

It is known that ethylene/1-hexene (EH) polymerization is also catalyzed by MgCl_2_/SiO_2_/TiCl_4_/THF-ZN catalyst in the slurry polymerization system using TEA as activator. However, the EH copolymerization with the catalyst activated by DEAC or TnHA has been found to exhibit different catalytic activity when compared to the system activated by TEA. To further explore the possibility of modifying catalytic activity and EH copolymer properties via changes in activator, TEA, DEAC, TnHA and their mixtures were employed in the equal molar ratio for each activator in the mixture. The Al/Ti molar ratio was kept constant at 300. The results are summarized in Table 2 and Figure 3. 

**Table 2:** Activity of ethylene/1-hexene copolymerization with various ratios of activatorsand % 1-hexene insertion.

Run number	Activator^a^ used			Activity (kg polymer/g cat/ h/ atm)	1-hexene insertion (mol%)^b^
	TEA (mol%)	DEAC (mol%)	TnHA (mol%)		
16	100	-	-	2.53	1.27
17	-	100	-	1.50	0.49
18	-	-	100	1.91	1.90
19	50	50	-	3.22	0.70
20	50	-	50	4.15	1.26
21	-	50	50	2.75	0.60
22	33	33	33	5.69	1.10

^a^ Ratio of Al/Ti =300; ^b^ 1-hexene insertion was determined by ^13^C-NMR.

**Figure 4:**
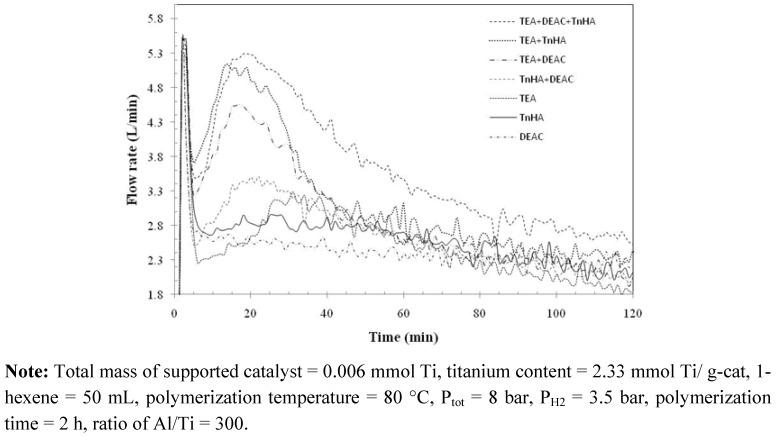
Kinetic profile based on ethylene consumption with various activators for ethylene/1-hexene copolymerization.

Interestingly, the kinetic profile behaviors of ethylene/1-hexene copolymerization with various activators were not a simple superposition of those activated by TEA, DEAC or TnHA alone, as shown in Figure 4. From this viewpoint, this result supports the notion of a change of alkyl groups in the alkyl aluminum mixture as mentioned before. From Table 2 and Figure 3, it was evident that the activator exerted strong influence on both catalytic activity and copolymer properties. The activity increased upon mixing TEA, DEAC and TnHA (TEA+DEAC, TEA+TnHA and TEA+DEAC+TnHA). The insertion of 1-hexene in copolymer increased in the order of TnHA > TEA > DEAC > mixed activators. No relationship between the catalytic activity and 1-hexene insertion was found with the different activators.

Many explanations have been put forth for the reported co-monomer effects on the enhancement of catalytic activity and can be classified as follows: (i) chemical and physical effects of co-monomer on the catalyst generation of active sites [37], (ii) increasing propagation rate constant (*kp*) [37], (iii) enhancement of the diffusion due to lower crystallinity [38], (iv) fracturing the catalyst, and (v) changing the oxidation state of Ti [38]. It seems that some of these explanations cannot possibly explain the mixed activator behavior, which should be concerned with the change in the active sites for the ZN catalyst with using different types of alkyl aluminum. Based on 1-hexene insertion, it seems that DEAC can effectively activate those active sites that produce partly crystalline copolymer, whereas the other activators such as TEA and TnHA were more efficient activators of active sites that produce amorphous copolymer. In the case of TEA+DEAC+TnHA mixture, it was able to activate both types of active sites, leading to a copolymerization system with high activity and slightly lower content of 1-hexene insertion. On the other hand, TnHA itself can produce a copolymer having relatively higher degree of 1-hexene insertion. 

### 2.4. Polymer characterization

#### 2.4.1. Gel permeation chromatography (GPC) analysis

The M_w_, M_n_ and MWD of the corresponding polymers are shown in Table 3. The MWD of the obtained polyethylene (Al/Ti ratio 300) gradually changed depending on the types of activator used. Apparently, TnHA produced polymers with a broader MWD as compared to polyethylene obtained from TEA and DEAC. Thus, the use of different types of activators in ethylene polymerization resulted in changes of the M_w_ and MWD values of polyethylene. The mixed alkyl aluminum system tended to exhibit a broad polyethylene MWD, as shown in Table 3. This result was also consistent with the dependence of MWD on the type of alkyl aluminum activator as shown in a previous report [24]. This interesting phenomenon was considered to stem from the existence of multiplicity in the nature of active sites with different propagation, termination and chain transfer rates on the surface of functionalized MgCl_2_/SiO_2_-supported ZN catalyst. According to the GPC profiles (not shown), it might be speculated that mainly two kinds of active titanium precursors exist on the surface of functionalized MgCl_2_/SiO_2_-supported ZN catalyst. The various alkyl aluminum activators may provide different types of active sites and different oxidation state of Ti. The M_w_, M_n_ and MWD results of the copolymers are also given in Table 3. The TEA, DEAC and TnHA gave M_w_ of 295, 364 and 336 kg/mol, and MWD of 3.7, 3.6 and 4.2, respectively. The average molecular weights of copolymers obtained from mixed activators tended to decrease compared to those obtained with a single activator. On the contrary, the MWD of copolymers increased as the follows: TEA+DEAC+TnHA (5.6) > TEA+TnHA (4.5) > TnHA (4.3) > TnHA+DEAC (3.8) = TnHA+DEAC (3.8) > TEA (3.7) > DEAC (3.6). These results showed no relationship among the M_w_, MWD, reducing power and activity in copolymerization system.

**Table 3:** M_W_, M_n_ and polydispersity (M_w_/M_n_) of polyethylene and ethylene/1-hexene copolymer.

Run	Monomer	Activator	M_n_ (kg/ mol)	M_w_ (kg/ mol)	M_w_/M_n_^a^	T_m_^b^ (°C)	d^c^ (g/mL)	Crystallinity (%)	
								DSC^d^	X-ray^e^
3	Ethylene	TEA	74	299	4.0	136.5	0.955	66.0	71.3
6		DEAC	51	180	3.5	136.3	0.957	67.7	-
9		TnHA	103	474	4.6	135.9	0.956	66.3	-
11		TEA+ DEAC	31	175	5.6	136.3	0.957	67.7	-
13		TEA+ TnHA	86	344	4.0	135.9	0.957	67.7	-
15		TEA+ DEAC+ TnHA	55	283	5.2	136.3	0.957	67.6	-
16	Ethylene/ 1-hexene	TEA	165	295	3.8	120.7	0.926	41.0	46.2
17		DEAC	56	364	6.5	124.4	0.943	55.5	60.1
18		TnHA	79	336	4.2	97.5	0.914	30.7	35.4
19		TEA+ DEAC	77	294	3.8	122.5	0.941	54.0	59.1
20		TEA+ TnHA	56	255	4.5	111.6	0.918	34.1	38.2
21		TnHA+ DEAC	54	209	3.8	123.7	0.943	55.3	60.3
22		TEA+ DEAC+ TnHA	48	272	5.6	119.5	0.923	38.4	42.2

^a ^Polydispersity index, evaluated as M_w_/M_n_, and determine by GPC analysis; ^b ^Melting temperature determined by DSC analysis; ^c ^Copolymer density determined according to the semi-empirical equation: d = (2195+△H_m_)/2500;^d^ Crystallinity degree determined according to the equation: (△H_m_/△H_m_°) × 100, assuming △H_m_° = 293 J/g; ^e ^Crystallinity degree determined by XRD according to the equation: W_c,x_ = (*I*_110_ + 1.42*I*_200_) / (*I*_110_ + 1.42*I*_200_ + 0.68*I*_a_).

#### 2.4.2. X-ray diffraction (XRD) and thermal properties

**Figure 5:**
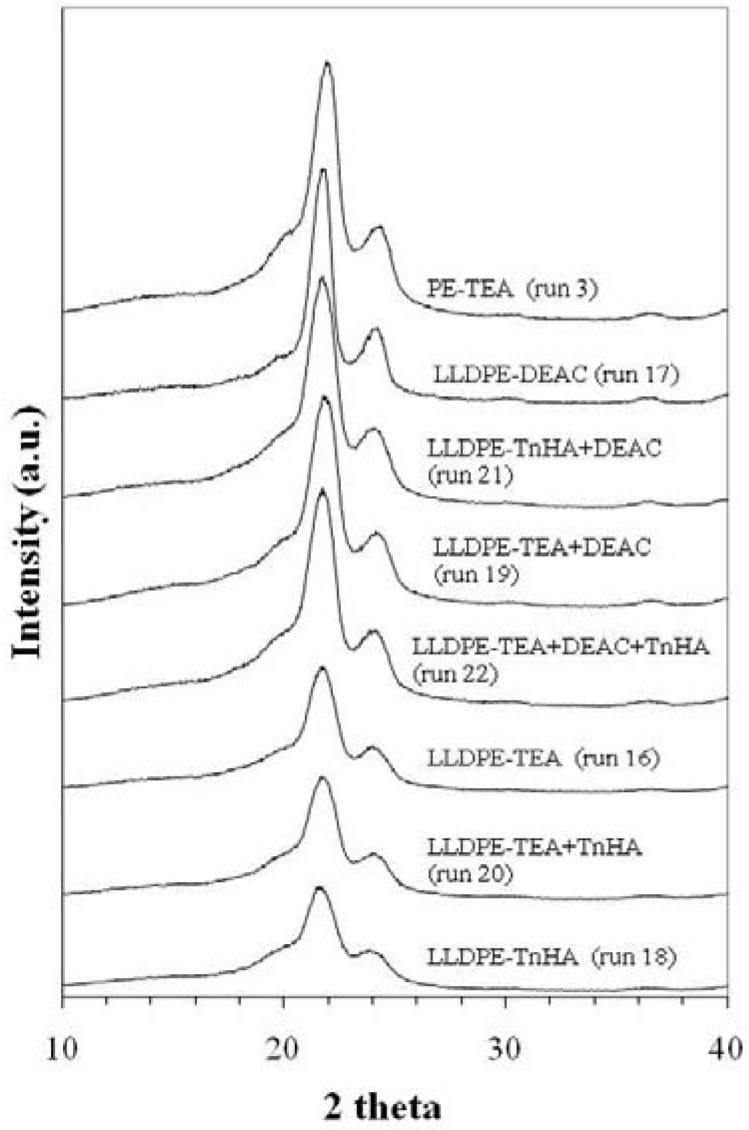
XRD patterns of PE and EH copolymers obtained with various activators.

It is well known that the melting enthalpy (△H_m_) of an EH copolymer decreases with increasing comonomer content [30]. The insertion of the α-olefin reduces both the degree of crystallinity and the melting temperature of the copolymer. Randall [39] found that for EH copolymer, the density of the sample decreases with increasing the comonomer content. Table 3 shows the melting temperatures of the EH copolymers obtained with different activators. It was very interesting that both melting temperature and enthalpy of melting decreased with TnHA. Figure 5 shows the XRD patterns of EH copolymers and homopolymer (PE) obtained with different activators. It was observed that the copolymers exhibit two crystalline peaks at 2θ degree of 21.1 and 23.58 assigned to 110 and 200 spacing and one amorphous peak at 2θ degree of 19.48. It was evident that the degree of crystallinity decreased with increasing comonomer content. The degrees of crystallinity calculated from both enthalpy and XRD are shown in Table 3. The results are very close to those reported by Mo *et al.* [14] and Quijada [1]. The values from XRD measurement are higher than those obtained from the DSC measurement. This can be attributed to different treatment of polymer samples prior to measurement for each technique. It can be accepted that different activators had no effect on the crystallinity of the polyethylene. However, in the case of EH copolymer, changes in activator can alter the crystallinity of copolymer due to different insertion of 1-hexene, as determined by the ^13^C-NMR. 

**Figure 6:**
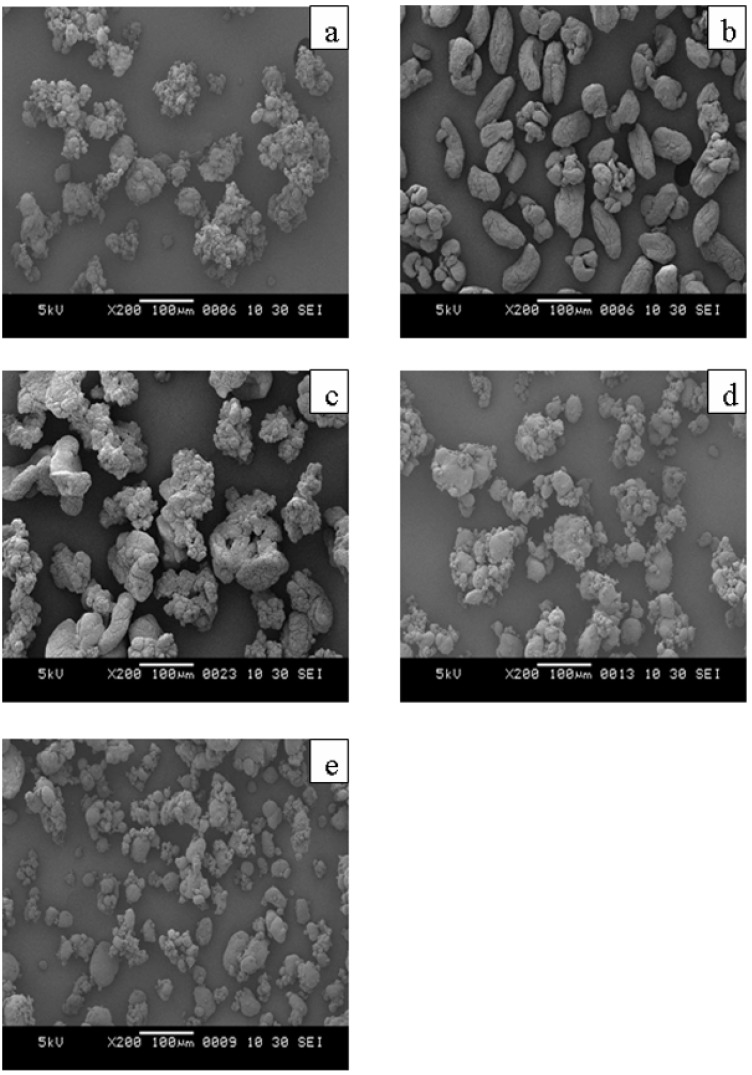
SEM micrographs of secondary product particles obtained with different activators; (a) TEA, (b) DEAC, (c) TnHA, (d) TEA+DEAC, (e) TEA+DEAC+TnHA.

Different polymerization systems produced different polyethylene particle shapes, as seen by SEM in Figure 6. These results may result from the different reducing powers of the activators. In addition, alkyl aluminums also participate in the termination of polymer chain growth, *i.e.* act as chain transfer agents, and/or reactivation of dormant sites [40]. 

2.4.3. ^13^C-NMR analysis

**Figure 7:**
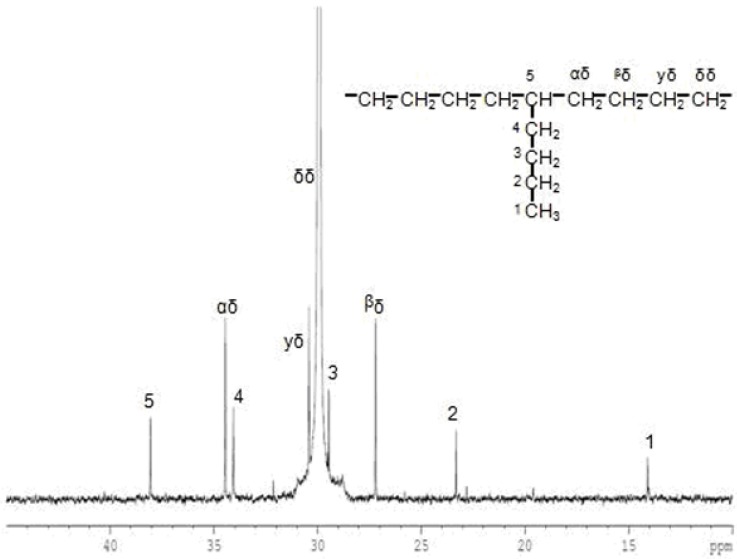
A typical ^13^C-NMR spectrum of EH copolymer obtained with TEA.

The incorporation of 1-hexene in copolymers was determined by ^13^C-NMR spectroscopy. The chemical dislocations in the copolymers were calculated according to the work of Randall [39]. Table 4 shows 1-hexene incorporation in EH copolymers with different activators in the polymerization system. It was found that copolymers had 1-hexene insertion in the range of 0.49-1.90 mol%, depending on the type of activator used. Apparently, DEAC produced copolymer having the lowest 1-hexene insertion (0.49 mol%). On the contrary, TnHA gave the highest 1-hexene insertion in the copolymer (1.90 mol%). It is worth noting that the alkyl aluminum mixtures such as TEA+DEAC and TnHA+DEAC produced copolymer having 1-hexene insertion values around 0.70 and 0.60 mol%, respectively. Similarly, TEA and TEA+TnHA had a 1-hexene insertion that was very close to that of TEA alone. It should be noted that 1-hexene unit was isolated by ethylene units, and no sequence of double co-monomer units or alternating ethylene/1-hexene units were found, as shown in Figure 7. The same results were also obtained with other activators. 

**Table 4:** Triad distribution based on ^13^C-NMR for EH copolymer obtained from different activators.

Run	Activator	[HHH]	[EHH]	[EHE]	[EEE]	[HEH]	[HEE]	%E	%H
16	TEA	0.0	0.7	0.6	97.0	0.2	1.2	98.73	1.27
17	DEAC	0.0	0.2	0.3	98.8	0.1	0.6	99.51	0.49
18	TnHA	0.0	1.2	0.7	96.3	0.4	1.4	98.10	1.90
19	TEA+DEAC	0.0	0.2	0.5	98.2	0.1	1.0	99.30	0.70
20	TEA+TnHA	0.0	0.7	0.6	97.2	0.2	1.2	98.74	1.26
21	TnHA+DEAC	0.0	0.2	0.4	98.4	0.2	0.8	99.40	0.60
22	TEA+DEAC+TnHA	0.0	0.4	0.7	97.4	0.1	1.4	98.89	1.11

## 3. Experimental

### 3.1. Chemicals

All reactions were performed under purified argon atmospheres using a standard glove box and Schlenk techniques. Polymerization grade ethylene, donated by the PTT Company, was used as received. Triethylaluminum (TEA), tri-*n*-hexylaluminum (TnHA), diethyl aluminum chloride (DEAC), donated by Tosoh Akzo Corp., were stored in a glove box and used without further purification. TiCl_4_ (Aldrich), MgCl_2_ (anhydrous) was donated by Tosoh Akzo Corp. Silica (specific surface area of 150 m^2^/g), supplied by Grace Davision, was heated under vacuum at 400 °C for 6 h. Hexane purchased from Aldrich Chemical Company Inc., was purified by passing it through a 13X molecular sieves column. Tetrahydrofuran and 1-hexene were dried over dehydrated CaCl_2_ and distilled over sodium/ benzophenone under an argon atmosphere prior to use. Ultra high purify (UHP) argon (99.999%) was purchased from Thai Industrial Gas Co., Ltd. and was further purified by passing through 3Å molecular sieves, BASF catalyst R3-11G, NaOH and phosphorus pentaoxide (P_2_O_5_) to remove traces of oxygen and moisture.

### 3.2. Catalyst preparation

The catalyst was prepared in a 500 mL vessel equipped with temperature control, and a turbine agitator. Anhydrous tetrahydrofuran (150 mL) was added to the vessel. The tetrahydrofuran was heated to 50 °C, and then magnesium metal (0.12 g) was added, followed by titanium tetrachloride (2 mL). The mixture was continuously agitated. The temperature was held at about 70 °C for 3 h. At the end of this time, magnesium dichloride (4.5 g) was added and heating was continued at 70 °C for another 3 h. Then, Grace Davision silica (4.5 g) was added over several minutes and the mixture was stirred for 1 h. This mixture was washed with hexane, and then dried under vacuum. The titanium content in the catalyst is 2.33 wt% (ICP).

### 3.3. Polymerization reaction

The slurry polymerization was performed in hexane solution (1,000 mL) with various activator mixtures as shown in Table 1 (Al/Ti molar ratio = 100 and 300). First hexane (1,000 mL) was added into the reactor under argon atmosphere. After that, activators and catalyst were introduced into a 2 L stainless steel reactor equipped with a magnetic stirrer, and the reactor was then evacuated to remove the argon. Hydrogen (P_H2_ = 3.5 bar) was fed into the reactor prior to the introduction of ethylene. The polymerization reaction was initiated by introducing the ethylene (P_C2H4_ = 4.5 bar). The total pressure in the reactor was 8 bar. The polymerization reaction was held constant at 80 °C and terminated after 2 h by quenching with dilute hydrochloric acid solution in methanol. The resulting polymer was isolated and dried under vacuum.

The EH copolymerization was performed as follows; the 2 L autoclave was evacuated and purged with argon several times similar to the ethylene polymerization. After hexane (950 mL) was added at 80 °C, the solution of activator such as TEA, DEAC, TnHA and TEA+DEAC+TnHA mixtures, 1-hexene (50 mL) and the catalyst (Al/Ti = 300) were added into reactor sequentially. Then, the 3.5 bar of hydrogen was fed into the reactor. The ethylene gas was fed into reactor and the total pressure in reactor was raised to 8 bar and held constant by continuous feed. After 2 h, the copolymerization was terminated using the same procedure as mentioned for ethylene polymerization. 

### 3.4. Polymer characterization

A high temperature GPC (PL-GPC220) equipped with a viscometric detector, differential optical refractometer and four Styragel HT type columns (HT3, HT4, HT5, and HT6) with a 1 × 10^7^ exclusion limit for polystyrene was used to determine the molecular weight (M_W_) and molecular weight distributions (MWD) of the polymers produced. The analyses were performed at 160 ºC using 1,2,4-trichlorobenzene as the solvent. The columns were calibrated with standard narrow MWD polystyrene. 

The comonomer contents of the resultant copolymers were determined by ^13^C-NMR spectroscopy. The measurements were performed at 110 °C on Bruker 400 MHz instrument using 1,2,4-trichlorobenzene as solvent. The thermal behavior of polyethylene and EH copolymers was examined with a Perkin-Elmer Pyris Diamond DSC at standard heating/cooling rate of 10 °C/min, under N_2_ flow. The samples were first melted to 180 °C (1st scan) and kept at this temperature for 3 min, then cooled to 50 °C, and again heated up to the melting with the same heating rate (2nd scan). The reported melting temperature values are referred to the second heating scan. The melting temperature (Tm) and the melting enthalpy (△H_m_) were taken from the second heating curve. Temperatures and heats of phase transitions were determined, respectively, from the maxima and areas of the crystallization and melting peaks. In this context, it was possible to relate △H_m_ (J/g) to the density (d, g/mL) of the copolymer through the following semiempirical equation: d = (2195 + △H_m_)/2500 [41]. Finally, using standards of known composition, a linear correlation between sample density and its content in terms of 1-hexene co-units was found, at least in the 0.92-0.94 g/mL density range [41]. The degree of crystallinity, X_c_ of PE and its copolymers was calculated from the ratio between the values of melting enthalpy, △H_m_ (as calculated from the second heating scan) and the heat of fusion of 100% crystalline PE taken as △H_m_ = 293 J/g [42].

X-ray diffraction patterns (XRD) analysis was carried out on a Siemens D-5000 apparatus working at 40 kV and 30 mA and using the Cu K_α_ radiation ( λ = 0.154439 Å) in the 10°-40° 2θ range with a scanning step of 0.01° in the reflection geometry. The crystalline degrees of the copolymers were calculated via Eq. (1) developed by Mo and Zhang [14]:

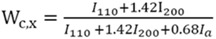
(1)


The morphological observations of polymers were carried out with a JEOL JSM-6400 scanning electron microscope (SEM). Micrographs were taken at a 5-kV acceleration voltage. Before SEM observations, the fracture surfaces of blends were coated with a thin layer of gold to avoid electrical charging and increase contrast during observation.

## 4. Conclusions

The use of suitable mixed activators such as TEA+DEAC+TnHA, can result in the significant increase in catalytic activity for the bi-supported Ziegler-Natta catalyst for ethylene polymerization and ethylene/1-hexene copolymerization. This can be attributed to the change in reducing power of the mixed activators leading to generation of different active forms of the catalyst or stabilization of the active center in ethylene polymerization. The activator type had an effect on the molecular weight and molecular weight distribution of polyethylene without any significant change in polymer morphology. In the case of copolymerization, there were no relationship among the M_w_, MWD and catalytic activity when the mixed activators were employed.

## References

[1] Quijada R., Guevara J.L., Galland G.B., Rabagliati F.M., Lopez-Majada J.M. (2005). Synthesis and properties coming from the copolymerization of propene with α-olefins using different metallocene catalysts. Polymer.

[2] Kawahara N., Kojoh S., Toda Y., Mizuno A., Kashiwa N. (2004). The detailed analysis of the vinylidene structure of metallocene-catalyzed polypropylene. Polymer.

[3] Cruz V., Ramos J., Munˇoz-Escalona A., Lafuente P., Penˇa B., Martinez S. (2004). 3D-QSAR analysis of metallocene-based catalysts used in ethylene polymerization. Polymer.

[4] Lee H.W., Chung J.S., Choi K.Y. (2005). Physical transitions and nascent morphology of syndiotactic polystyrene in slurry polymerization with embedded Cp*Ti(OMe)3/methyl aluminoxane catalyst. Polymer.

[5] Nitta K.H., Shin Y.W., Hashiguchi H., Tanimoto S., Terano T. (2005). Morphology and mechanical properties in the binary blends of isotactic polypropylene and novel propylene-co-olefin random copolymers with isotactic propylene sequence 1. Ethylene-propylene copolymers. Polymer.

[6] Wooster T.J., Abrol S., Macfarlane D.R. (2004). Cyanate ester polymerization catalysis by layered-silicates. Polymer.

[7] Chen S., Hua Z.J., Fang Z., Qi G.R. (2004). Copolymerization of carbon dioxide and propylene oxide with highly effective zinc hexacyanocobaltate(III)-based coordination catalyst. Polymer.

[8] Bazzini C., Giarrusso A., Porri L., Pirozzi B., Napolitano R. (2004). Synthesis and characterization of syndiotactic 3,4-polyisoprene prepared with diethylbis(2,2'-bipyridine)iron-MAO. Polymer.

[9] Liu J.Y., Zheng Y., Li Y.S. (2004). Polymerization of methyl methacrylate by iron(II) pyridinebisimine complexes. Polymer.

[10] Gao M.Z., Liu H.T., Wang J., Li C.X., Ma J., Wei G.S. (2004). Novel MgCl_2_-supported catalyst containing diol dibenzoate donor for propylene polymerization. Polymer.

[11] Kawahara N., Kojoh S.I., Toda Y., Mizuno A., Kashiwa N. (2004). The detailed analysis of the vinylidene structure of metallocene-catalyzed polypropylene. Polymer.

[12] Lee T.S., Kim J.W., Bae J.Y. (2004). Palladium-catalyzed selective dehalogenative homocoupling polymerization of AB2-type dihaloaryl sulfonate monomers. Polymer.

[13] Galland G.B., Quijada R., Rojas R., Bazan G., Zachary Komon J.A. (2002). NMR Study of Branched Polyethylenes Obtained with Combined Fe and Zr Catalysts. Macromolecules.

[14] Mo Z.S., Zhang H.F. (1995). The degree of crystallinity in polymers by wide-angle x-ray diffraction (WAXD). Macromol. Chem. Phys..

[15] Madri S., Xuejing Z., RoBert B.J., JOHN C.C., COR E.K. (2006). Effect of 1-Hexene Comonomer on Polyethylene Particle Growth and Copolymer Chemical Composition Distribution. J. Polym. Sci.: Part A: Polym. Chem..

[16] Yong P.C., Zhi F. (2006). Ethylene/1-hexene copolymerization with TiCl4/MgCl2/AlCl3 catalyst in the presence of hydrogen. Eur. Polym. J..

[17] Nooijen G.A.H. (1994). On the importance of diffusion of cocatalyst molecules through heterogeneous ziegler/natta catalysts. Eur. Polym. J..

[18] Lynch T.D., Jejelowo M.O., Wanke S.E. (1991). The influence of aluminum alkyls on the polymerization of ethylene with silica/magnesium chloride-supported titanium tetrachloride catalysts. Can. J. Chem. Eng..

[19] Siokou A., Ntais S. (2003). Towards the preparation of realistic model Ziegler-Natta catalysts: XPS study of the MgCl_2_/TiCl_4_ interaction with flat SiO_2_/Si(1 0 0). Surf. Sci..

[20] Dong Q., Fu Z., Xu J., Fan Z. (2007). Strong influences of cocatalyst on ethylene/propylene copolymerization with a MgCl_2_/SiO_2_/TiCl_4_/diester type Ziegler-Natta catalyst. Eur. Polym. J..

[21] Lynch D.T., Wanke S.E. (1991). Reactor Design and Operation for Gas-Phase Ethylene Polymerization Using Ziegler-Natta Catalysts. Can. J. Chem. Eng..

[22] Hammawa H., Mannan T.M., Lynch D.T., Wanke S.E. (2004). Effects of aluminum alkyls on ethylene/1-hexene polymerization with supported metallocene/MAO catalysts in the gas phase. J. Appl. Polym. Sci..

[23] Fukuda K., Liu B., Nakatani H., Nishiyama I., Yamahiro M., Terano M. (2003). Significant variation of molecular weight distribution (MWD) of polyethylene induced by different alkyl-Al co-catalysts using a novel surface functionalized SiO2-supported Ziegler-Natta catalyst. Catal. Commun..

[24] Haward R.N., Roper A.N., Fletcher K.L. (1973). Highly active catalysts for ethylene polymerization by the reduction of TiCl_4_ with organomagnesium compounds. Polymer.

[25] Gardner K., Parsons I.W., Haward R.N. (1978). Polymerization of propene with organomagnesium-reduced titanium (IV) chloride-based catalyst. J. Polym. Sci.: Polym. Chem..

[26] Kashiwa N. (1980). Super active catalyst for olefin polymerization. Polymer.

[27] Licchelli J.A., Haward R.N., Parsons I.W., Caunt A.D. (1981). Polymerization catalysts for propene from the reduction of titanium tetrachloride with halogen-free magnesium alkyls. Polymer.

[28] Machon J.P., Hermant R., Houzeaux J.P. (1975). Study of the catalytic activity of violet titanium trichloride in the high-temperature polymerization of ethylene. J. Polym. Sci. Symp. Ser..

[29] Munoz E.A., Hernandez J.G., Gallardo J.A., Keii T., Soga K. (1986). Design of supported Ziegler-Natta catalysts using silica as carrier. Stud. Surf. Sci. Catal..

[30] Munoz E.A., Garcia H., Albornoz A. (1987). Homo- and copolymerization of ethylene with highly active catalysts based on titanium tetrachloride and Grignard compounds. J. Appl. Polym. Sci..

[31] Zakharov V.A., Bukatov G.D., Ermakov Y. (1980). The Mechanism of the Catalytic Polymerisation of Olefins Based on the Number of Active Centres and the Rate Constants for Individual Stages. Russ. Chem. Rev..

[32] Mori H., Ohnishi K., Terano M. (1996). Ethene polymerization with modified-polypropene-supported highly stable Ziegler catalyst. Macromol. Rapid Commun..

[33] Zacca J.J., Debling J.A., Ray W.H. (1996). Reactor residence time distribution effects on the multistage polymerization of olefins - I. Basic principles and illustrative examples, polypropylene. Chem. Eng. Sci..

[34] Kim I., Chung M.C., Choi H.K., Kim J.H., Woo S.I. (1990). (1990) Homo- and Co-polymerization of Ethylene with the Highly Active TiCl_4_/THF/MgCl_2_ Catalyst. Catal. Olefin Polym..

[35] Kim I., Kim J.H., Woo S.I. (1990). Kinetic Study of Ethylene Polymerization by Highly Active Silica Supported TiCl_2_ MgCl_2_ Catalyst. J. Appl. Polym. Sci..

[36] Hatada K., Yuki H. (1967). Alkyl interchange in the mixture of triethyl aluminum and diethylaluminum chloride. Tetrahedron Lett..

[37] Calabro D.C., Lo F.Y., Quirk R.P. (1988). A comparison of the reaction kinetics for the homo- and copolymerization of ethylene and hexene with a heterogeneous Ziegler catalys. Transition Metal Cata lyzed Polymerizations: Ziegler–Natta and Methathesis Polymerization.

[38] Chien J.C.W., Nozaki T. (1993). Ethylene-hexene copolymerization by heterogeneous and homogeneous Ziegler-Natta catalysts and the "comonomer" effect. J. Polym. Sci..

[39] Randall J.C. (1989). A review of high-resolution liquid carbon-13 nuclear magnetic resonance characterizations of ethylene-based polymers. AJMS-REV. Macromol. Chem. Phys..

[40] Zakharov V.A., Bukatov G.D., Yermakov Y.I. (1983). On the mechanism of olefin polymerization by Ziegler-Natta catalysts. Adv. Polym. Sci..

[41] Carlini C., Alessio A.D., Giaiacopi S., Po R., Pracella M., Galletti A.M.R., Sbrana G. (2007). Linear low-density polyethylenes by co-polymerization of ethylene with 1-hexene in the presence of titanium precursors and organoaluminium co-catalysts. Polymer..

[42] Wunderlich B., Czornyj G. (1977). A study of equilibrium melting of polyethylene. Macromolecules.

